# A Novel Tetravalent CD95/Fas Fusion Protein With Superior CD95L/FasL Antagonism

**DOI:** 10.1002/prot.26741

**Published:** 2024-09-01

**Authors:** Isabell Lang, Oliver Paulus, Olena Zaitseva, Harald Wajant

**Affiliations:** ^1^ Department of Internal Medicine II, Division of Molecular Internal Medicine University Hospital Würzburg Wurzburg Germany

**Keywords:** apoptosis, CD95/Fas, CD95L/FasL, protein engineering, protein–protein interaction

## Abstract

Inhibition of CD95/Fas activation is currently under clinical investigation as a therapy for glioblastoma multiforme and preclinical studies suggest that disruption of the CD95–CD95L interaction could also be a strategy to treat inflammatory and neurodegenerative disorders. Besides neutralizing anti‐CD95L/FasL antibodies, mainly CD95ed‐Fc, a dimeric Fc fusion protein of the extracellular domain of CD95 (CD95ed), is used to prevent CD95 activation. In view of the fact that full CD95 activation requires CD95L‐induced CD95 trimerization and clustering of the resulting liganded CD95 trimers, we investigated whether fusion proteins of the extracellular domain of CD95 with a higher valency than CD95ed‐Fc have an improved CD95L‐neutralization capacity. We evaluated an IgG1(N297A)‐based tetravalent CD95ed fusion protein which was obtained by replacing the variable domains of IgG1(N297A) with CD95ed (CD95ed‐IgG1(N297A)) and a hexavalent variant obtained by fusion of CD95ed with a TNC‐Fc(DANA) scaffold (CD95ed‐TNC‐Fc(DANA)) promoting hexamerization. The established N297A and DANA mutations were used to minimize FcγR binding of the constructs under maintenance of neonatal Fc receptor (FcRn) binding. Size exclusion high‐performance liquid chromatography indicated effective assembly of CD95ed‐IgG1(N297A). More important, CD95ed‐IgG1(N297A) was much more efficient than CD95ed‐Fc in protecting cells from cell death induction by human and murine CD95L. Surprisingly, despite its hexavalent structure, CD95ed‐TNC‐Fc(DANA) displayed an at best minor improvement of the capacity to neutralize CD95L suggesting that besides valency, other factors, such as spatial organization and agility of the CD95ed domains, play also a role in neutralization of CD95L trimers by CD95ed fusion proteins. More studies are now required to evaluate the superior CD95L‐neutralizing capacity of CD95ed‐IgG1(N297A) in vivo.

## 1 Introduction

CD95 (Fas, Apo‐1, TNFRSF6) is a member of the tumor necrosis factor (TNF) receptor superfamily (TNFRSF) and contains in its ectodomain three cysteine‐rich domains (CRDs), the structural characteristic of this protein family [1]. CD95 is activated by CD95L. Furthermore, CD95 contains a cytoplasmic death domain (DD), a protein–protein interaction domain enabling the recruitment of cytosolic death domain‐containing proteins by DD–DD interactions in response to CD95 stimulation by CD95L. By virtue of its interaction with the DD‐containing adapter protein Fas‐associated death domain (FADD), CD95 recruits procaspase‐8 and promotes it's processing to mature heterotetrameric caspase‐8 which in turn can trigger apoptosis. By the help of the DD‐containing kinase RIPK1, CD95 can also stimulate necroptotic cell death. CD95, again by help of FADD, caspase‐8 and RIPK1, can also stimulate proinflammatory signaling and calcium signaling [2, 3].

CD95L is a major effector molecule of activated T cells and CD95‐mediated cell death accordingly plays a crucial role in tumor surveillance. Noteworthy, CD95 and CD95L are also involved in the downregulation of immune responses by the elimination of activated T cells and autoreactive B cells. Deregulated excessive CD95‐mediated cell death can also have severe consequences for health. For example, CD95‐mediated cell death signaling is responsible for LPS‐induced lung injury and contributes to influenza A‐induced pneumonia, GvHD, and diabetes [4, 5, 6, 7, 8, 9]. Furthermore, defects in CD95 or CD95L result in the autoimmune lymphoproliferative syndrome (ALPS) and can promote tumorigenesis [10, 11]. Worth mentioning, CD95‐resistant tumors may exploit the non‐cell death signaling capabilities of CD95 to promote proliferation and cell migration of tumor cells and CD95L expression to escape the immune system [10].

In line with the pathophysiological role of the CD95L–CD95 system, biologicals interfering with the interaction of CD95L and CD95 revealed therapeutic activity in various preclinical disease models. Indeed, Asunercept (APG101, CAN008), a bivalent fusion protein of the CD95 ectodomain and the Fc domain of human IgG1, is currently under investigation in a clinical phase II trial to treat patients with newly diagnosed glioblastoma (ClinicalTrials.gov Identifier: NCT05447195).

Here, we show that CD95ed‐IgG1(N297A), a tetravalent CD95ed fusion protein, generated by replacing the variable domains of IgG1(N297A) with CD95ed, which can be considered as a CD95ed dimer of dimer, has superior CD95L‐inhibitory activity compared with CD95ed‐Fc.

## 2 Materials and Methods

### 2.1 Cell Lines and Reagents

HEK293T, HT1080, Jurkat, and A20J cells were cultivated in RPMI1640 medium (Sigma‐Aldrich, Germany) supplemented with 10% fetal calf serum (GIBCO). Jurkat Rapo and Jurkat Rapo‐CD95L cells have already been described elsewhere [12]. Antibodies were purchased from the following suppliers: Sigma‐Aldrich, St. Louis, Missouri (anti‐Flag M2, #F‐3165; anti‐β‐actin #A1978‐200; horseradish peroxidase [HRP] coupled anti‐rabbit antibody, #7074), BD Biosciences, NJ (anti‐PARP, #551025), Cell Signaling, MA (anti‐Caspase‐9, #9502; anti‐Caspase‐3 #8G10; anti‐IκBα #L35A5; anti‐P‐IκBα #14D4), Enzo life sciences (anti‐Caspase‐8 #ADI‐AAM‐118‐E) and Dako, Glostrup, Denmark ([HRP]‐conjugated polyclonal rabbit anti‐mouse antibody, #P0260). Expression plasmids encoding for the following proteins were obtained from Origene (Rockville, Maryland): Human FcRn (#RC200364), murine FcRn (MG#205688), and beta‐2 microglobulin (#RC207587).

### 2.2 Production and Purification of CD95 Ectodomain (CD95ed) Fusion Proteins

The various CD95ed fusion proteins were produced using HEK293T cells upon transient transfection with the corresponding construct encoding expression plasmids or 1:1 expression plasmid mixtures (CD95ed‐IgG1(N297A) and CD95ed‐IgG1(N297A)‐LC:GpL) using polyethylenimine (PEI, Polyscience Inc., Warrington, Pennsylvania) as described elsewhere for other proteins [13]. The amino acid sequences of the encoded proteins are listed in the Table S1. Cell culture supernatants of transfected cells were collected after 5–7 days, cleared by centrifugation for 10 min (4630*g*) and the fusion protein concentrations were estimated by western blotting with the anti‐Flag mAb M2. For this purpose, the intensities of the western blot signals of the Flag‐tagged CD95ed fusion proteins were compared with those of a serial dilution of a Flag‐tagged standard protein of known concentration separated on the same gel. The Flag‐tagged proteins were purified by affinity chromatography on anti‐Flag M2 agarose and Flag peptide as eluent according to the manufacturer's protocol (Sigma‐Aldrich, Steinheim, Germany). The purity of the proteins was analyzed by SDS‐PAGE and subsequent silver staining of the gel using the Pierce Silver Stain Kit (Thermo Fisher Scientific, Massachusetts). The concentration of the purified proteins was defined by comparison with the proteins of known size and concentration of the “Low Molecular Weight Calibration Kit for SDS Electrophoresis” (GE Healthcare UK Limited, Little Chalfont, UK) which were coapplied on the gel.

### 2.3 High Performance Liquid Chromatography (HPLC)

To check for potential aggregation of recombinant proteins, the affinity‐purified protein samples were analyzed by gel filtration on a MabPac SEC‐1 column (Thermo Fisher, #088460) in PBS with a flow rate of 0.76 mL/min using the UltiMate 3000 HPLC system (Thermo Fisher). As size and quality standards the biologicals Enbrel (TNFR2‐Fc) and Humira (anti‐TNF IgG1) were analyzed under the same conditions.

### 2.4 Binding Studies

The interactions of the CD95ed fusion proteins with CD95L were analyzed in cell‐free binding studies with *Gaussia princeps* luciferase (GpL) fusion proteins of the various CD95ed variants. Black high‐binding 96‐well plates were coated overnight at 4°C with either 2 μg/mL Fc‐Flag‐CD95L or 2 μg/mL TNFR2‐Fc in 0.1 M carbonate buffer. Next day, wells were washed three times with PBST and were then blocked with 10% FCS in PBS for 1 h. The various CD95ed‐GpL fusion proteins were added pairwise with increasing concentrations to the TNFR2‐Fc and Fc‐Flag‐CD95L coated wells for 1 h at 37°C. After three additional washing steps with PBST, wells were washed 5–10 times with ice cold PBS to remove unbound proteins and then 50 μL RPMI1640 with 0.5% FCS and 1% Pen/Strep were added. Finally, GpL activity was determined by adding 25 μL GpL substrate solution (1.5 μM Coelentarazine [Carl Roth, Karlsruhe, Germany] in PBS) per well and measuring the luminescence signal (LUmo luminometer, anthos Mikrosysteme GmbH, Friesoythe, Germany). Specific binding values were calculated by subtraction of the unspecific binding values derived of TNFR2‐Fc coated wells from the total binding values obtained from the corresponding Fc‐Flag‐CD95L coated wells.

The interactions of CD95ed‐IgG1(N297A) with the human and murine FcRn were analyzed with cellular binding studies and a GpL‐linked variant of CD95ed‐IgG1(N297A). In brief, HEK293T cells were transiently transfected using the PEI method [13] with a mixture (1:1) of expression plasmids encoding beta‐2 microglobulin and the human or murine FcRn to determine total binding. HEK293T cells transfected in parallel with empty vector (EV) served furthermore to determine unspecific binding. Next day FcRn‐ and EV‐transfected cells were harvested and distributed into 1.5 mL tubes (1 × 10^6^ cells per tube). The FcRn‐ and EV‐transfected cells were then pairwise incubated with increasing concentrations of CD95ed‐IgG1(N297A)‐LC:GpL for 1 h at 37°C in RPMI 1640 medium with 10% FCS adjusted to pH value 5.5. Unbound proteins were removed by three washing steps (1 min centrifugation, 14 000 rpm) with icecold PBS (pH 5.5). Finally, cell pellets were resuspended in 50 μL RPMI1640 with 0.5% FCS and 1% Pen/Strep, transferred to black 96‐well plates and GpL activity was measured again as described above for the cell‐free binding studies. Specific binding values were again obtained by subtraction of the unspecific binding values from the matched total binding values.

To obtain KD‐values the specific binding values were fit to a single binding site type of interaction using the non‐linear regression analysis function of the GraphPad Prism 5 software.

### 2.5 Viability Assay

The ability of the various CD95ed fusion proteins to inhibit CD95L‐induced cell death was evaluated using HT1080, Jurkat and A20J cells. Cells were cultivated overnight in 96 well cell culture plates (HT1080: 2 × 10^4^ per well, Jurkat: 4 × 10^4^ per well, A20J: 8 × 10^4^ per well). Cells were then treated with mixtures of the various CD95ed fusion proteins and Fc‐Flag‐CD95L or Fc‐Flag‐TNC‐muCD95L and after an additional day, cellular viability was quantified by crystal violet staining (HT1080) or using the MTT assay (Jurkat, A20J). HT1080 and A20J cells were sensitized for cell death‐induction by adding 2.5 μg/mL cycloheximide (CHX) 30 min prior adding the biological combinations. Values for 100% and 0% cell viability for normalization were obtained from untreated cells (or, where appropriate, CHX treated cells) and cells challenged with a highly cytotoxic mixture of 1 μg/mL Fc‐Flag‐CD95L and 5 μg/mL CHX ensuring complete cell killing. To evaluate the inhibitory effect of the CD95ed fusion proteins on membrane CD95L‐induced cell death, HT1080 cells were seeded in 96 well cell culture plates (2 × 10^4^) and cultivated overnight. Next day, HT1080 cells were sensitized with 2.5 μg/mL CHX for 30 min and stimulated with mixtures of 37 500 Jurkat Rapo‐CD95L cells and 5 μg/mL of the CD95ed fusion proteins or 37 500 Jurkat Rapo cells (CD95L negative) only (to define 100% HT1080 viability). Rapo cells are a Jurkat variant, which is CD95/Fas deficient and Rapo‐CD95L is a stable transfectant generated thereof expressing memCD95L/memFasL and has been described elsewhere [12]. Cellular viability of HT1080 cells was quantified the next day by crystal violet staining.

### 2.6 ELISA

The ability of the CD95ed fusion proteins to inhibit memCD95L‐induced stimulation of IL8 production was determined in cocultures of HT1080 and Jurkat Rapo‐CD95L cells. HT1080 cells (2 × 10^4^ per well) were seeded overnight in 96 well cell culture plates. Next day, Jurkat Rapo or Jurkat Rapo‐CD95L suspension cells (5 × 10^4^ per well) were preincubated with 10 μg/mL of CD95ed‐Flag‐Fc or CD95ed‐Flag‐IgG1(N297A) or remained untreated. Then the cell culture medium of HT1080 cells was replaced with fresh medium containing 20 μM of the pan‐caspase inhibitor zVAD (Bachem, Weil am Rhein, Germany). After additional 30 min, Jurkat Rapo and Jurkat Rapo‐CD95L cells mixed with the indicated CD95ed fusion proteins were added. Next day, supernatants of the cocultures were analyzed for the presence of IL8 using the BD OptEIA TM human IL8‐ELISA kit (BD Biosciences, New Jersey). IL8 production of HT1080 cells mixed with Jurkat Rapo cells has been defined as 0% and IL8 production of HT1080 cells mixed with Jurkat Rapo‐CD95L has been defined 100% IL8‐induction.

### 2.7 Western Blotting

Cells were seeded in 6‐well plates overnight (HT1080 1.5 × 10^6^) or the same day (Jurkat 2 × 10^6^) and then cells were stimulated for 4 h with mixtures of the various CD95ed fusion proteins and Fc‐Flag‐CD95L as indicated in the figure legends. To prepare total cell lysates, cells were harvested with a rubber policeman and/or by centrifugation. Cell pellets were resuspended in 4× Laemmli sample buffer (8% SDS, 10% β‐mercaptoethanol, 40% glycerol, 0.2 M Tris, pH 8.0) supplemented with phosphatase inhibitor mixture II (Sigma Aldrich. Steinheim, Germany). After sonification (30 s, UP100H Ultrasonic Processor, Helscher, Germany), samples were boiled at 95°C for 5 min and applied to SDS‐PAGE separation. Proteins were transferred to a 0.2 μM nitrocellulose membrane (Amersham, Freiburg, Germany) and after blocking with 5% non‐fat‐dried milk in TBS‐Tween, western blot analysis was performed with the primary antibodies of interest and corresponding secondary antibodies. Finally, antigen–antibody complexes were visualized using the ECL western blot detection system (Amersham, Freiburg, Germany).

### 2.8 Statistical Analyses and Software

Statistical analyses were performed by using the GraphPad Prism 5 software (GraphPadSoftware Inc., LaJolla, California).

## 3 Results

### 3.1 Valency Improves the Binding of the Soluble Ectodomain of CD95 to CD95L

**FIGURE 1:**
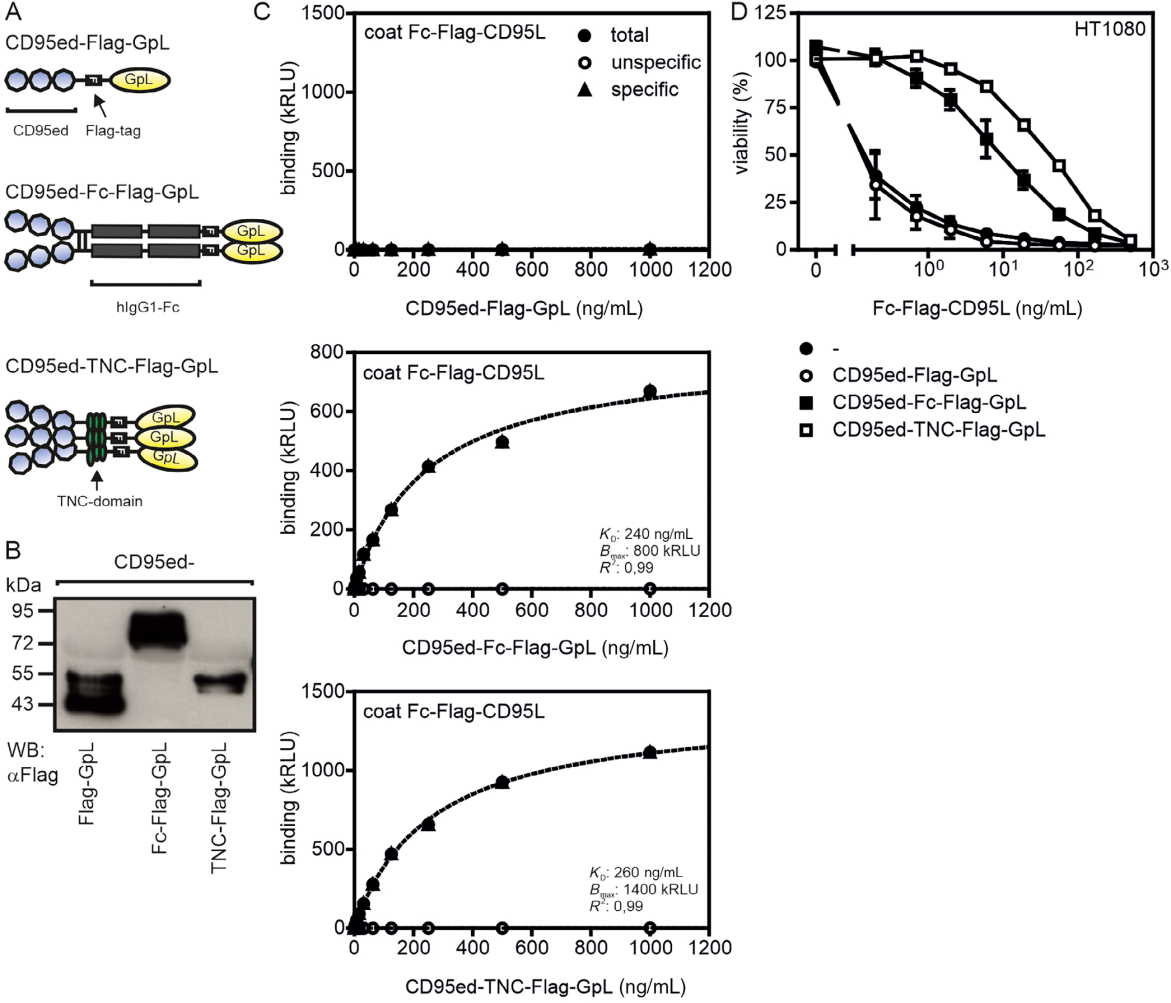
Valency is a crucial factor determining the affinity of CD95ed fusion proteins for CD95L. (A) Scheme of CD95ed GpL fusion proteins. (B) Western blot analysis of CD95ed GpL fusion proteins. (C) Specific binding of CD95ed‐GpL fusion proteins to plastic immobilized Fc‐CD95L. (D) Inhibition of Fc‐CD95L‐induced apoptosis of HT1080 cells by CD95ed‐GpL fusion proteins. 1 μg/mL of the indicated variants of CD95ed were preincubated with increasing concentrations of Fc‐CD95L for 30 min and then added to HT1080 cells sensitized with 2.5 μg/mL cycloheximide. After 18 h cell viability was determined by crystal violet staining.

Initially, we evaluated the relevance of valency for binding of the CD95 ectodomain (CD95ed) to CD95L. Binding studies with monomeric, dimeric and trimeric variants of CD95ed were performed. Di‐ and trimerization of CD95ed were enforced by genetic fusion of a Fc dimerization domain and the tenascin trimerization domain (TNC) to the C‐terminus of CD95ed. To allow easy and sensitive quantification of the CD95ed variants, CD95ed, CD95ed‐Fc and CD95ed‐TNC were furthermore linked with a C‐terminal *Gaussia princeps* luciferase (GpL) reporter domain (Figure 1A,B). Equilibrium binding studies with plastic‐immobilized Fc‐CD95L showed that CD95ed‐GpL failed to bind to CD95L while the dimeric and trimeric CD95ed‐GpL variants showed high affinity binding (Figure 1C). Similarly, CD95ed‐GpL failed to block Fc‐CD95L‐induced apoptosis of HT1080 cells while CD95ed‐Fc‐GpL and even better CD95ed‐TNC‐GpL inhibited Fc‐CD95L‐induced cell death (Figure 1D).

The lack of CD95L binding by the monomeric CD95ed version was striking. We therefore evaluated whether this was possibly due to misfolding of the CD95ed construct or assembly to inactive molecule aggregates. Indeed, there is evidence that CD95 dimerizes via its N‐terminal CRD, designated as pre‐ligand assembly domain (PLAD), in the absence of ligand without triggering CD95 signaling [14, 15, 16]. In gel filtration analysis of supernatants containing ~2–10 μg/mL CD95ed‐GpL, the latter eluted with a peak volume <50 kDa and thus gave no evidence for aggregation (Figure S1). In comparison, CD95‐TNC‐GpL eluted in fractions of a *M*_W_ >150 kDa (Figure S1). Dimerization of the isolated CD95ed by the PLAD domain seems therefore unlikely at the concentrations used in our study. Indeed, PLAD‐PLAD interactions are probably weak. For example, KD values of ~150 μg/mL were found for the PLAD autoaffinity of the PLAD of TNFR1 and TNFR2 in cell‐free experiments [17].

**FIGURE 2:**
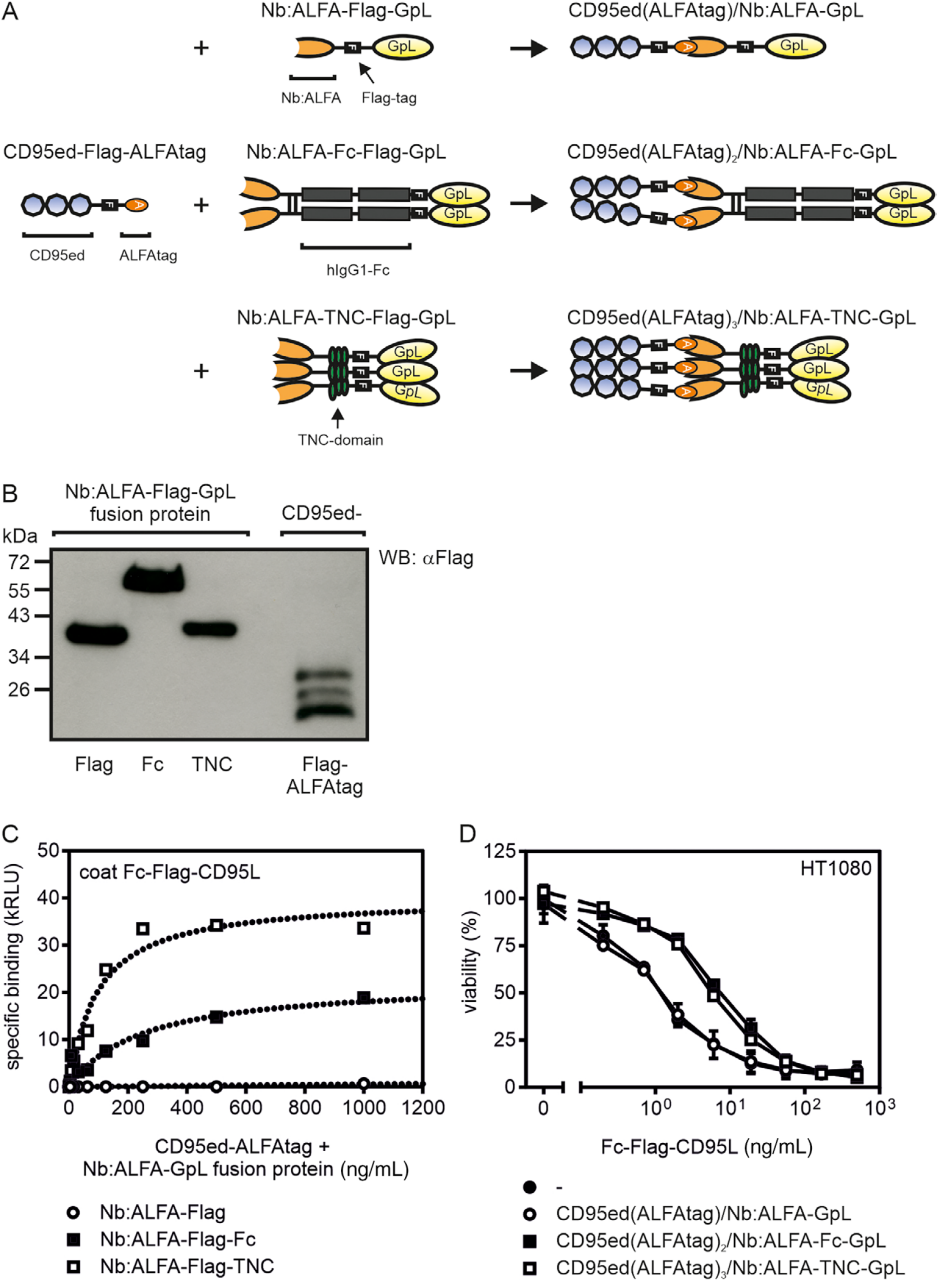
Di‐ and trimerization of monomeric CD95ed‐ALFAtag enable CD95L binding and inhibition of CD95L‐induced apoptosis. (A) Scheme of CD95ed‐ALFAtag and mono‐, di‐, and trivalent ALFAtag‐specific nanobody (Nb:ALFA) GpL fusion proteins. (B) Western blot analysis of SNs containing the proteins shown in A. Please note that CD95 is *N*‐glycosylated in its extracellular domain. This posttranslational modification leads to different molecular masses and thus the CD95ed–ALFAtag fusion protein appears in three distinct bands. (C) Specific binding of mixed SNs containing CD95ed–ALFAtag and mono‐, bi‐, and trivalent Nb:ALFA GpL fusion proteins in the ratio 2:1 to plastic immobilized Fc‐CD95L. (D) Inhibition of Fc‐CD95L‐induced apoptosis of HT1080 cells by mixed SNs containing 1 μg/mL ALFAtag CD95ed and 0.5 μg/mL of the mono‐, bi‐, or trivalent Nb:ALFA GpL fusion protein. Mixed supernatants were preincubated with increasing concentrations of Fc‐CD95L for 30 min and then added to HT1080 cells sensitized with 2.5 μg/mL cycloheximide. After 18 h cell viability was determined by crystal violet staining.

To evaluate whether the monomeric CD95ed misfolds, we produced CD95ed with a C‐terminal ALFAtag [18]. The resulting CD95ed(ALFAtag)‐containing supernatants were then mixed with supernatants containing monomeric, dimeric (Fc), or trimeric (TNC) GpL fusion proteins of a high‐affinity ALFAtag‐specific nanobody (Nb:ALFA) (Figure 2A,B). The mixed supernatants containing among others “bi‐molecular” mono‐, bi‐, and trivalent CD95ed(ALFAtag)/Nb:ALFA‐GpL complexes were then again analyzed with respect to CD95L binding and inhibition of CD95L‐induced cell death. The monovalent CD95ed(ALFAtag)/Nb:ALFA‐GpL complex‐containing SN mixtures showed no binding to CD95L and also no inhibitory effect on CD95L‐induced cell death (Figure 2C,D). The SN mixtures containing bi‐ and trivalent complexes of CD95ed (CD95ed(ALFAtag)_2_/Nb:ALFA‐Fc‐GpL and CD95ed(ALFAtag)_3_/Nb:ALFA‐TNC‐GpL), however, which were generated with the same CD95ed(ALFAtag) SN, showed good binding to CD95L and inhibition of CD95L‐induced apoptosis (Figure 2C,D). Apparently, the CD95ed alone is expressed as a correctly folded protein but is not able to bind CD95L strongly in this form.

### 3.2 Tetravalent CD95ed‐IgG1(N297A) Has a Higher CD95L‐Blocking Activity Than Bivalent CD95ed‐Fc

**FIGURE 3:**
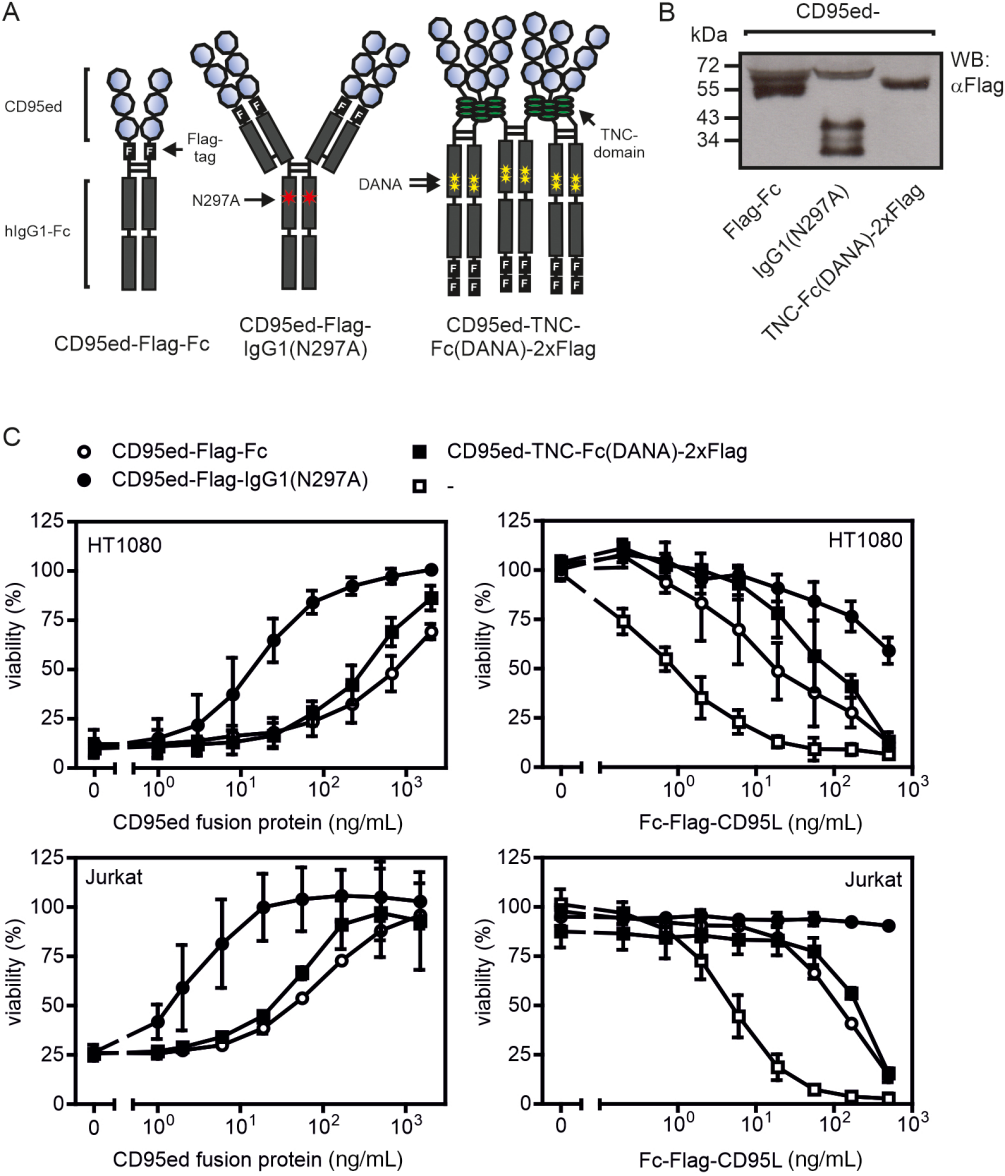
CD95ed‐IgG1(N297A) has superior antagonistic activity. (A) Scheme of domain architecture of di‐, tetra‐, and hexavalent fusion proteins of CD95ed. (B) Western Blot analysis of recombinant proteins. (C) Inhibition of Fc‐CD95L‐induced apoptosis of HT1080 and Jurkat cells. Left panel: Increasing concentrations of the indicated variants of CD95ed were preincubated with 10 ng/mL of Fc‐CD95L for 30 min and then added to cycloheximide sensitized HT1080 cells or Jurkat cells. After 18 h cell viability was determined by crystal violet staining (HT1080) or the MTT assay (Jurkat). Right panel: Increasing concentrations of Fc‐CD95L were preincubated with 1 μg/mL of the various CD95ed variants for 30 min and then added to cycloheximide sensitized HT1080 cells or Jurkat cells. After 18 h cell viability was again determined by crystal violet staining (HT1080) or the MTT assay (Jurkat).

We hypothesized that CD95ed fusion proteins with a higher valency than CD95ed‐Fc, which structurally corresponds to Asunercept (APG101, CAN008), have superior CD95L‐neutralizing activity. To test this, we generated tetra‐ and hexavalent CD95ed variants, which contained two dimeric or trimeric CD95ed subdomains, for comparison with CD95ed‐Fc as benchmark. To obtain a molecule containing two dimeric subdomains of CD95ed, we replaced the variable domains of an IgG1(N297A) antibody by CD95ed (Figure 3A,B). The N297A mutation present in the molecule is established in the field of antibody engineering and strongly reduces binding to FcγRs [19] and served to avoid potentially disturbing protein–protein interactions not related to the CD95L–CD95 interaction. To obtain a hexavalent molecule containing two trimeric subdomains of CD95ed, we replaced the GpL domain in the trimeric CD95ed‐TNC‐GpL variant by a human Fc IgG1 domain carrying the DANA mutations [20] which similar to the N297A mutation reduce interaction with FcγRs (Figure 3A,B). While cell culture supernatants (SNs) containing CD95ed‐Fc and CD95ed‐TNC‐Fc(DANA) showed a largely comparable capacity to inhibit cell death induction by Fc‐CD95L in HT1080 and Jurkat cells, CD95ed‐IgG1(N297A) SNs revealed a much higher capacity to inhibit Fc‐CD95L‐induced cell killing (Figure 3C).

### 3.3 Purification and Characterization of CD95(ed)‐IgG1(N297A)

**FIGURE 4:**
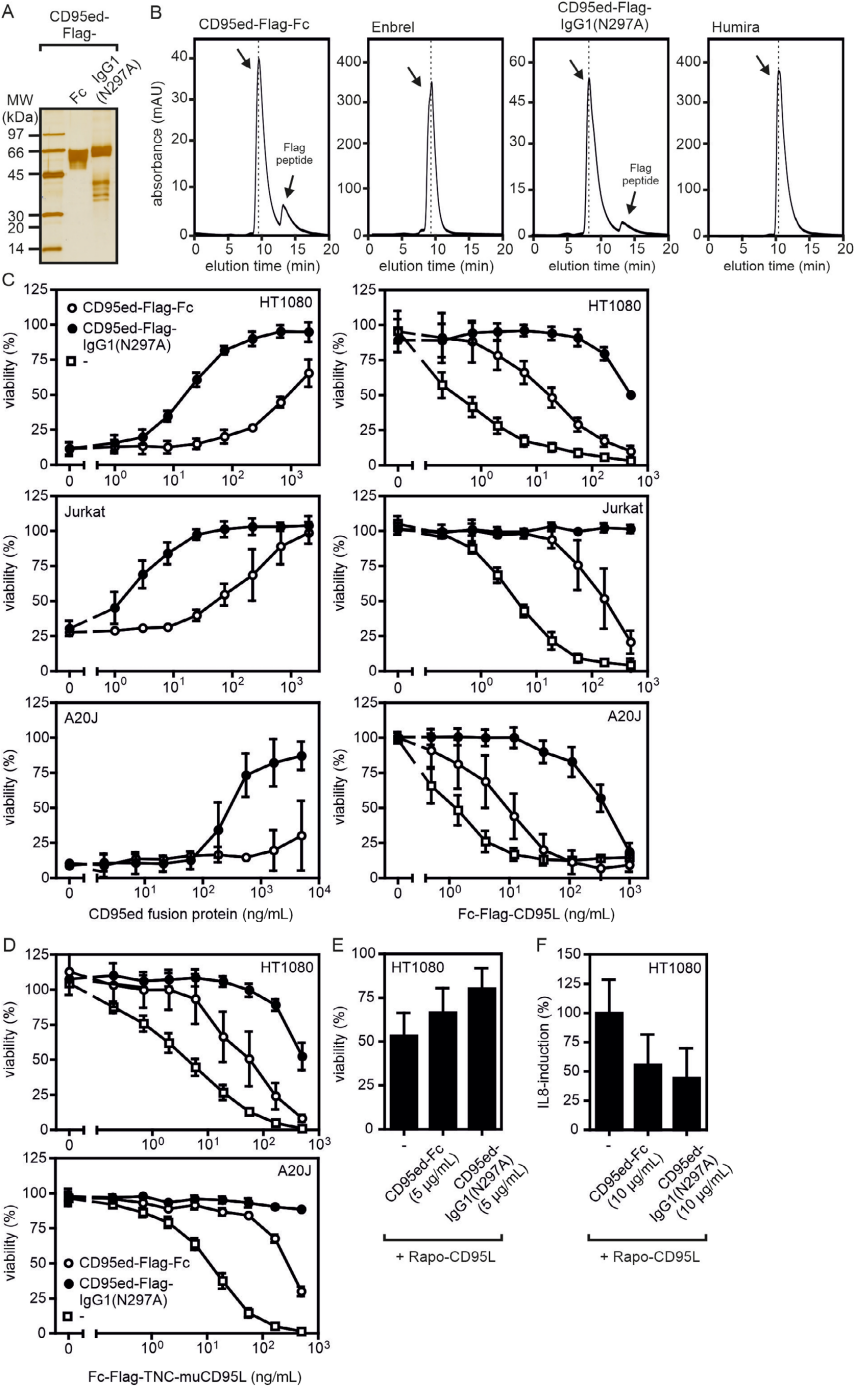
Biochemical characterization of CD95ed‐IgG1(N297A). (A) Anti‐Flag affinity purified CD95ed‐Fc and CD95ed‐IgG1(N297A) were separated by SDS‐PAGE and visualized by silver staining of the gel. (B) Purified proteins were analyzed by gel filtration in PBS on a MabPac SEC‐1 column with a flow rate of 0.76 mL/min. Enbrel (=TNFR2‐Fc, glycosylated *M*_w_ = 150 kDa) and Humira (anti‐TNF‐IgG1, *M*_w_ = 150 kDa) were analyzed by gel filtration, too, as high‐quality benchmarks of structurally related molecules. (C) Inhibition of human CD95L‐induced cell death by Fc‐CD95ed and CD95ed‐IgG1(N297A). Please note, HT1080 and Jurkat are human cell lines while A20J is a murine cell line. Left panel: Increasing concentrations of the indicated variants of CD95ed were preincubated with 10 ng/mL (50 ng/mL for A20J cells) of Fc‐CD95L for 30 min and then added to cycloheximide sensitized HT1080 cells or Jurkat or A20J cells. After 18 h cell viability was determined by crystal violet staining (HT1080) or the MTT assay (Jurkat, A20J). Right panel: Increasing concentrations of Fc‐CD95L were preincubated with 1 μg/mL (5 μg/mL for A20J cells) of the various CD95ed variants for 30 min and then added to cycloheximide sensitized HT1080 cells or Jurkat or A20J cells. After 18 h cell viability was again determined by crystal violet staining (HT1080) or the MTT assay (Jurkat, A20J). (D) Inhibition of murine CD95L‐induced cell death by Fc‐CD95ed and CD95ed‐IgG1(N297A). Increasing concentrations of Fc‐TNC‐muCD95L were preincubated with 1 μg/mL (5 μg/mL for A20J cells) of the various CD95ed variants for 30 min and then added to cycloheximide sensitized HT1080 cells or A20J cells. After 18 h cell viability was again determined by crystal violet staining (HT1080) or the MTT assay (A20J). (E,F) Inhibition of induction of apoptosis (E) and IL8 production (F) by membrane CD95L expressing cells by the indicated CD95ed variants (10 μg/mL) in cocultures of HT1080 cells and Jurkat Rapo‐CD95L cells. Please note, to prevent cell death‐related effects, apoptosis induction was prevented by cotreatment with 20 μm ZVAD in (F).

We purified CD95ed‐IgG1(N297A) and CD95ed‐Fc for further analyses by affinity chromatography exploiting the Flag tag present in both molecules. Both CD95ed variants were efficiently purified this way (Figure 4A). Gel filtration analysis furthermore confirmed correct and uniform assembly of the two proteins (Figure 4B). CD95ed‐Fc eluted with 9.5 min and thus with the same size as Enbrel (TNFR2‐Fc) which we analyzed as a clinical grade TNFR‐Fc fusion protein benchmark (Figure 4B). CD95ed‐IgG1(N297A), which is 30 kDa larger than an IgG1 antibody, eluted furthermore faster than the clinical used anti‐TNF IgG1 antibody Humira which we used as IgG1 gel filtration benchmark (Figure 4B). Most important, purified CD95ed‐IgG1(N297A) was again clearly superior compared with CD95ed‐Fc with respect to inhibition of Fc‐CD95L‐induced caspase activation and cell killing in HT1080 and Jurkat cells (Figures 4C and S2). Similarly, CD95ed‐IgG1(N297A) was also superior to CD95ed‐Fc with respect to Fc‐CD95L‐induced killing of the murine cell line A20J and murine CD95L‐induced cell death (Figure 4C,D). Not unexpected, CD95ed‐IgG1(N297A) was also superior to CD95ed‐Fc in inhibition of memCD95L‐induced cell death of HT1080 cells and preventing CD95‐mediated non‐cell death signaling resulting, for example, in the production of IL8 (Figure 4E,F).

### 3.4 CD95ed‐IgG1(N297A) Interacts With FcRns

**FIGURE 5:**
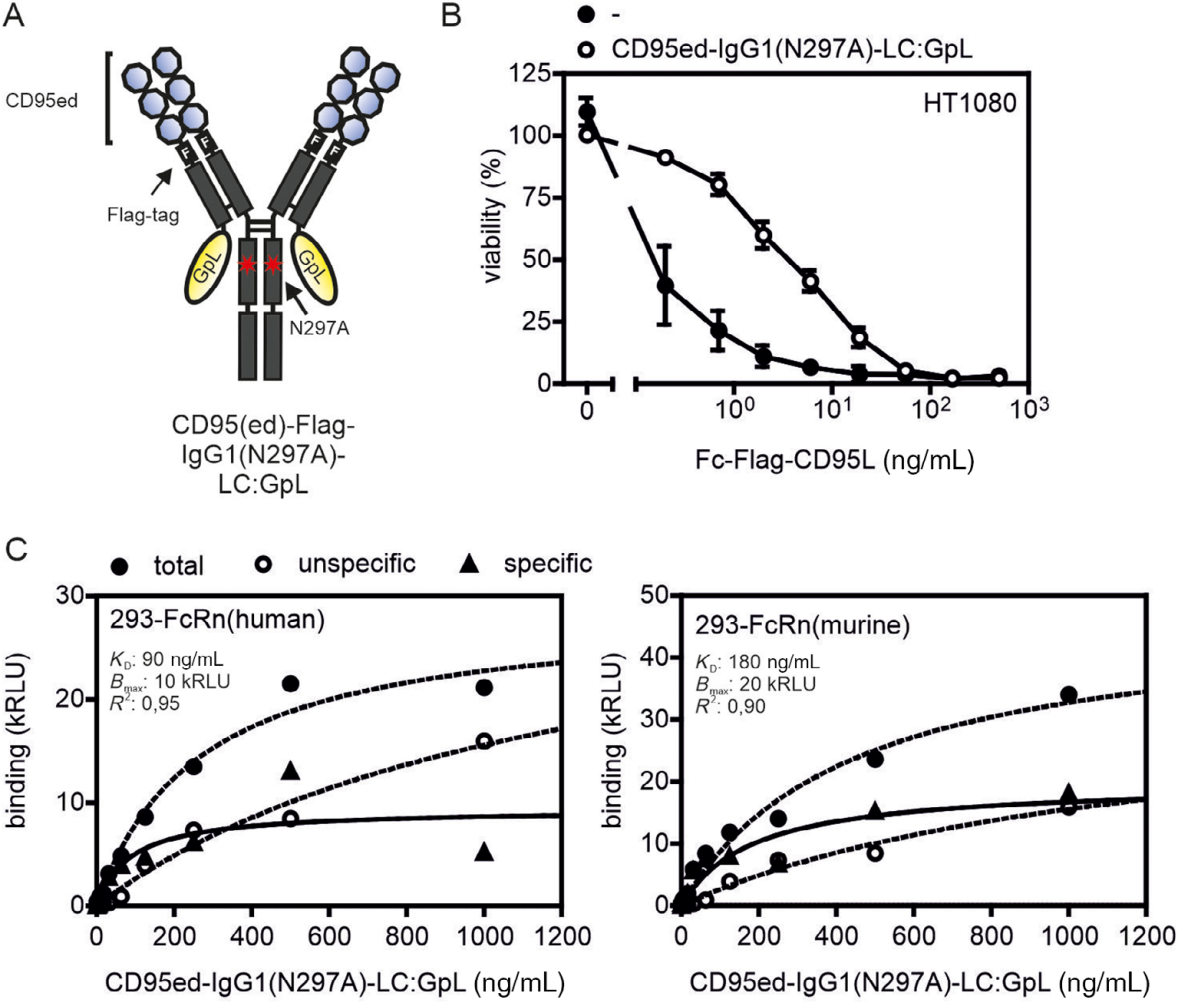
CD95ed‐IgG1(N297A) interacts with FcRns. (A) Scheme of domain architecture of CD95ed‐IgG1(N297A)‐LC:GpL. (B) Inhibition of apoptosis induced by increasing concentrations of Fc‐CD95L by 1 μg/mL of CD95ed‐IgG1(N297A)‐LC:GpL in HT1080 cells. (C) Specific binding of CD95ed‐IgG1(N297A)‐LC:GpL to HEK293 cells transfected with human and murine FcRn.

For effective in vivo activity of a ligand neutralizing biological not only the specific capacity to bind a ligand and to hinder its interaction with its receptor(s) is important but also its retention in the circulation. The latter is typically particular good for immunoglobulins and Fc fusion proteins due to the interaction with neonatal FcRn. To control that this important interaction is maintained in CD95ed‐IgG1(N297A) as expected from the literature [19], we analyzed a variant of the molecule with a GpL domain at the C‐terminus of the CL domain for FcRn binding (Figure 5A). Inhibition of Fc‐CD95L‐induced apoptosis of HT1080 cells proved that the modified molecule is still an effective CD95L inhibitor (Figure 5B). Most important, however, CD95ed‐IgG1(N297A)‐LC:GpL interacted with high affinity with human and murine FcRn (Figure 5C). Thus, CD95ed‐IgG1(N297A) promises similar good serum retention as conventional N297A mutated IgG1 molecules.

## 4 Discussion

**FIGURE 6:**
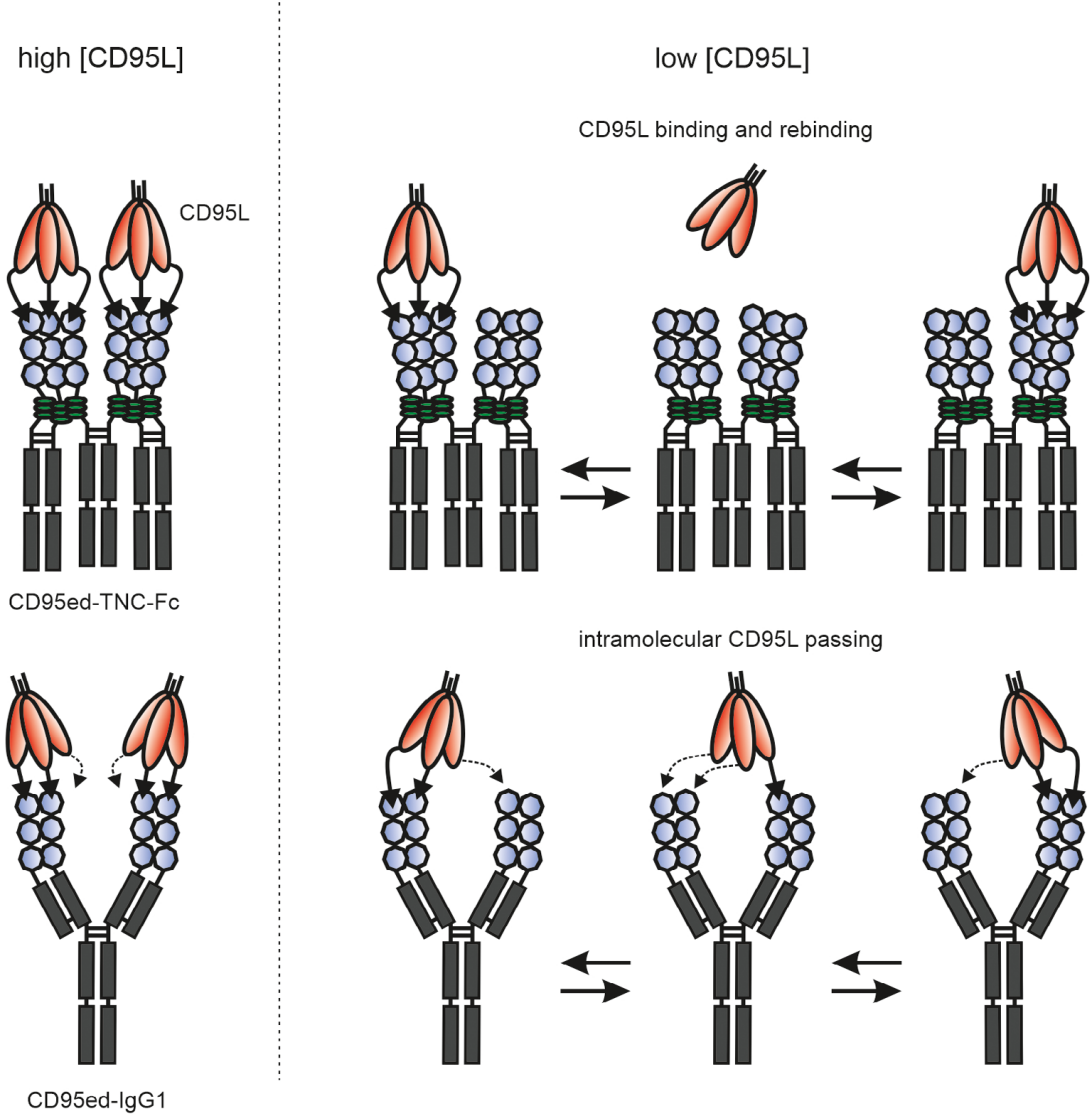
Proposed mode of action of CD95ed‐IgG1(N297A) and CD95ed‐TNC‐Fc. For details see main text. Please note, CD95ed dimers and CD95ed trimers have largely the same affinity for CD95L trimers (see Figure 1C).

Trimeric CD95ed and dimeric CD95ed elicited practically similar affinities for CD95L (Figure 1C). Accordingly, despite its higher valency CD95ed‐TNC was at best moderately more effective in neutralization of CD95L‐induced cell killing than CD95ed‐Fc (Figure 1D). The clearly superior capacity of the tetravalent CD95ed‐IgG1(N297A) construct to block CD95L‐induced signaling in comparison to the bivalent benchmark CD95ed‐Fc and especially in comparison to the hexavalent CD95ed‐TNC‐Fc is therefore surprising. A possible explanation for the counterintuitive superior inhibitory activity of the tetravalent CD95ed‐IgG1(N297A) construct could lie in how the CD95ed domains are assembled in the construct in detail. In the trimeric construct CD95ed‐TNC as well as in the hexameric construct CD95ed‐TNC‐Fc, the CD95ed domain is followed by the 29 aa coiled‐coil domain from the tenascin‐C molecule, which forms a covalently bound very compact parallel trimer [21]. It therefore seems plausible that CD95ed‐TNC effectively forms a parallel‐oriented trimer that saturates the three receptor binding sites of a CD95L trimer (Figure 6). Accordingly, the two trimeric CD95ed‐TNC subdomains of the hexameric CD95ed‐TNC‐Fc fusion protein should independently bind CD95L trimers. In particular, due to the stiff structural organization the three CD95ed domains of a CD95ed‐TNC subdomain, it appears possible that these three CD95ed domains have to interact with the same CD95L trimer. Thus, CD95ed domains located in different CD95ed‐TNC subdomains of the molecule might not be able to bind the same CD95L trimer. Therefore, at lower concentrations of CD95L when only one CD95ed‐TNC subdomain is occupied by a CD95L trimer, the latter might not be simply passed to the non‐occupied CD95ed‐TNC subdomains by mixed interactions of the CD95eds of the two subdomains (Figure 6). The situation might, however, be different in the case of the tetravalent CD95ed‐IgG1(N297A) molecule. Here, as in CD95ed‐TNC‐Fc, are two parallel‐oriented CD95L‐binding CD95ed subdomains, which, analogous to the formation of a Fab subdomain in an IgG antibody, should form between the CD95ed‐CL and the CD95ed‐CH1 domain of the construct. However, the two CD95ed‐CL/CH1 subdomains are only bivalent and therefore bind a CD95L trimer in a non‐saturating manner (Figure 6). Furthermore, the two CD95ed‐CL/CH1 subdomains in the CD95ed‐IgG1(N297A) molecule are by far less rigid and aligned in parallel than the two CD95L‐binding subdomains in the CD95ed‐TNC‐Fc molecule due to the indirect connection via the flexible hinge region of the antibody scaffold. It, therefore, seems possible that at low concentrations, when only one of the two bivalent CD95L binding subdomains of CD95ed‐IgG1(N297A) has bound a CD95L trimer, the free second CD95ed‐CL/CH1 subdomain also makes an interaction with the remaining free binding site of the CD95L trimer. After release from the first bipartite subdomain this binding mode may then result to facilitated bivalent rebinding via the still monovalently connected second subdomain. This intramolecular “CD95L passing” mode of CD95L trimer binding of CD95ed‐IgG1(N297A), however, is currently fully speculative and must be evaluated in future studies.

## Author Contributions

**Isabell Lang:** conceptualization, writing – original draft, visualization, investigation, methodology. **Oliver Paulus:** methodology, investigation. **Olena Zaitseva:** methodology, investigation. **Harald Wajant:** conceptualization, writing – review and editing, project administration, supervision.

## Supporting information


**Data S1.** Supporting Information.

## Data Availability

The data that support the findings of this study are available from the corresponding author upon reasonable request.
