# Supplementary material for: A Novel Tetravalent CD95/Fas Fusion Protein With Superior CD95L/FasL Antagonism
Source: Proteins. 2024 Sep 1;93(2):441–51. doi: 10.1002/prot.26741 (PMC11694555; doi:10.1002/prot.26741)
Supplement: Supplementary file 1 — Data S1. Supporting Information. [file PROT-93-441-s001.docx]

**Supplemental Data**

**A novel tetravalent CD95/Fas fusion protein with superior CD95L/FasL antagonism**

Isabell Lang^1^, Oliver Paulus, Olena Zaitseva, and Harald Wajant


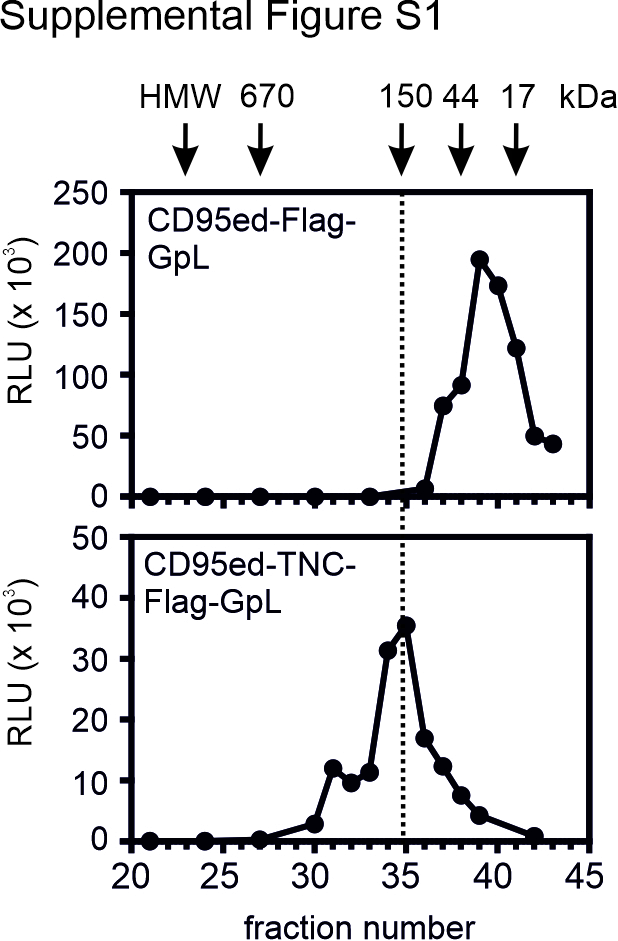


Supplemental Figure S1. **Gel filtration analysis of CD95ed-GpL and CD95ed-TNC-GpL.** Cell culture supernatants containing CD95ed-GpL and CD95ed-TNC-GpL were separated on a BioSep-SEC-S3000 (300×7.8) column (Phenomenex, Aschaffenburg, Germany) equilibrated with PBS (flow rate of 0.5 ml/min). Collected fractions were then diluted 1:5 in RPMI1640 with 0.5 % FCS and 1% Pen/Strep and 50 µl were then transferred to black 96-well plates to measure GpL activity as described in the materials and methods section. A marker protein mixture (Column Performance Check Standard, Aqueous SEC 1; Phenomenex, Aschaffenburg, Germany) containing thyroglobulin (670 kDa), human immunoglobulin G (150 kDa), ovalbumin (44 kDa) and myoglobulin (17 kDa) was analyzed in parallel. The peak positions of the marker proteins are indicated.


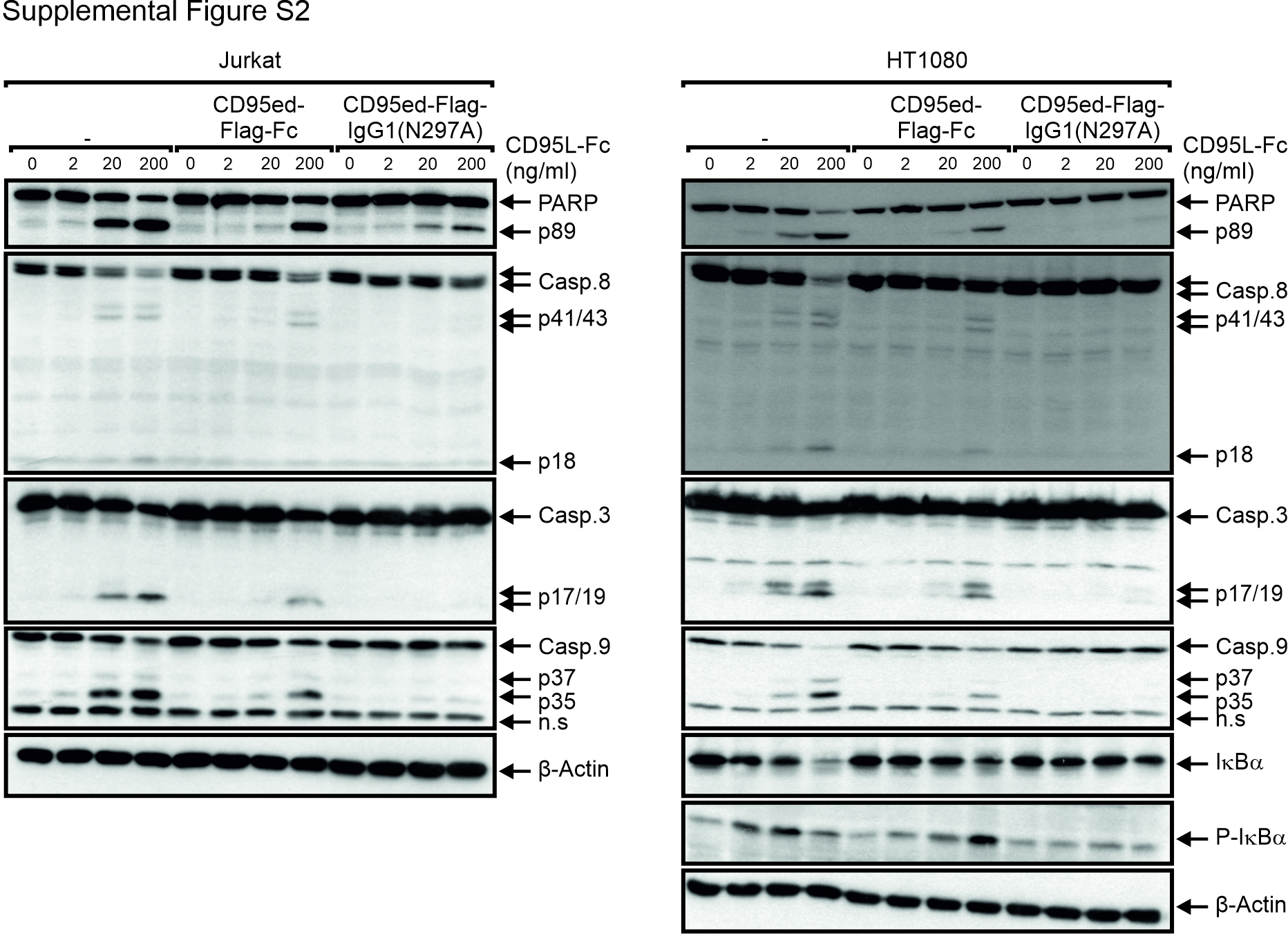


Supplemental Figure S2. **Inhibition of CD95L-induced caspase activation.** Jurkat and HT1080 cells were challenged with the indicated concentrations of Fc-CD95L along with 1 µg/ml CD95ed-Fc or CD95ed-IgG1(N297A) or were only treated with Fc-CD95L in the presence of 2.5 µg/ml CHX. Next day, total cell lysates were analyzed for the presence of the indicated proteins by Western blotting.

Supplemental Table S1. **Amino acid sequences of encoded proteins.**

| # | Name pCR3 based expression plasmid | Corresponding amino acid sequence |
| --- | --- | --- |
|  | Legend |  |
| 1 | CD95ed-Flag-GpL-pCR3 | MDWTWRVFCLLAVAPGAHSLE*VARLSSKSVNA*QVTDINSKGLELRKTVTTVETQNLEGLHHDGQFCHKPCPPGERKARDCTVNGDEPDCVPCQEGKEYTDKAHFSSKCRRCRLCDEGHGLEVEINCTRTQNTKCRCKPNFFCNSTVCEHCDPCTKCEHGIIKECTLTSNTKCKEEGSRSNGS**DYKDDDDK**EFALKPRLEKPTENNEDFNIVAVASNFATTDLDADRGKLPGKKLPLEVLKEMEANARKAGCTRGCLICLSHIKCTPKMKKFIPGRCHTYEGDKESAQGGIGEAIVDIPEIPGFKDLEPMEQFIAQVDLCVDCTTGCLKGLANVQCSDLLKKWLPQRCATFASKIQGQVDKIKGAGGD |
|  | Leader Ig heavy chain V-I region HG3, CD95(ed): CRD1, CRD2, CRD3, cleavage site, **Flag-tag**, GpL |  |
| 2 | CD95ed-Flag-Fc-pCR3 | *MLGIWTLLPLVLTSVARLSSKSVNA*QVTDINSKGLELRKTVTTVETQNLEGLHHDGQFCHKPCPPGERKARDCTVNGDEPDCVPCQEGKEYTDKAHFSSKCRRCRLCDEGHGLEVEINCTRTQNTKCRCKPNFFCNSTVCEHCDPCTKCEHGIIKECTLTSNTKCKEEGSRSNEF**DYKDDDDK**LEKTHTCPPCPAPELLGGPSVFLFPPKPKDTLMISRTPEVTCVVVDVSHEDPEVKFNWYVDGVEVHNAKTKPREEQYNSTYRVVSVLTVLHQDWLNGKEYKCKVSNKALPAPIEKTISKAKGQPREPQVYTLPPSRDELTKNQVSLTCLVKGFYPSDIAVEWESNGQPENNYKTTPPVLDSDGSFFLYSKLTVDKSRWQQGNVFSCSVMHEALHNHYTQKSLSLSPGK |
|  | CD95(ed): *Leader*, CRD1, CRD2, CRD3, **Flag-tag**, cleavage site, hIgG1-Fc |  |
| 3 | CD95ed-Fc-Flag-GpL-pCR3 | MDWTWRVFCLLAVAPGAHSLE*VARLSSKSVNA*QVTDINSKGLELRKTVTTVETQNLEGLHHDGQFCHKPCPPGERKARDCTVNGDEPDCVPCQEGKEYTDKAHFSSKCRRCRLCDEGHGLEVEINCTRTQNTKCRCKPNFFCNSTVCEHCDPCTKCEHGIIKECTLTSNTKCKEEGSRSNGSKTHTCPPCPAPELLGGPSVFLFPPKPKDTLMISRTPEVTCVVVDVSHEDPEVKFNWYVDGVEVHNAKTKPREEQYNSTYRVVSVLTVLHQDWLNGKEYKCKVSNKALPAPIEKTISKAKGQPREPQVYTLPPSRDELTKNQVSLTCLVKGFYPSDIAVEWESNGQPENNYKTTPPVLDSDGSFFLYSKLTVDKSRWQQGNVFSCSVMHEALHNHYTQKSLSLSPGKEF**DYKDDDDK**LEKPTENNEDFNIVAVASNFATTDLDADRGKLPGKKLPLEVLKEMEANARKAGCTRGCLICLSHIKCTPKMKKFIPGRCHTYEGDKESAQGGIGEAIVDIPEIPGFKDLEPMEQFIAQVDLCVDCTTGCLKGLANVQCSDLLKKWLPQRCATFASKIQGQVDKIKGAGGD |
|  | Leader Ig heavy chain V-I region HG3, CD95(ed): CRD1, CRD2, CRD3, cleavage site, hIgG1-Fc, **Flag-tag**, GpL |  |
| 4 | CD95ed-TNC-Flag-GpL-pCR3 | MDWTWRVFCLLAVAPGAHSLEVARLSSKSVNAQVTDINSKGLELRKTVTTVETQNLEGLHHDGQFCHKPCPPGERKARDCTVNGDEPDCVPCQEGKEYTDKAHFSSKCRRCRLCDEGHGLEVEINCTRTQNTKCRCKPNFFCNSTVCEHCDPCTKCEHGIIKECTLTSNTKCKEEGSRSNPRDIACGCAAAPDIKDLLSRLEELEGLVSSLREQGTGGGSGRGS**DYKDDDDK**EFALKPRLEKPTENNEDFNIVAVASNFATTDLDADRGKLPGKKLPLEVLKEMEANARKAGCTRGCLICLSHIKCTPKMKKFIPGRCHTYEGDKESAQGGIGEAIVDIPEIPGFKDLEPMEQFIAQVDLCVDCTTGCLKGLANVQCSDLLKKWLPQRCATFASKIQGQVDKIKGAGGD |
|  | Leader Ig heavy chain V-I region HG3, CD95(ed): CRD1, CRD2, CRD3, cleavage site, TNC, **Flag-tag**, GpL |  |
| 5 | CD95ed-Flag-CH1-CH3(N297A)-pCR3 | MDWTWRVFCLLAVAPGAHSLE*VARLSSKSVNA*QVTDINSKGLELRKTVTTVETQNLEGLHHDGQFCHKPCPPGERKARDCTVNGDEPDCVPCQEGKEYTDKAHFSSKCRRCRLCDEGHGLEVEINCTRTQNTKCRCKPNFFCNSTVCEHCDPCTKCEHGIIKECTLTSNTKCKEEGSRSNGS**DYKDDDDK**EFSSASTKGPSVFPLAPSSKSTSGGTAALGCLVKDYFPEPVTVSWNSGALTSGVHTFPAVLQSSGLYSLSSVVTVPSSSLGTQTYICNVNHKPSNTKVDKKVEPKSCDKTHTCPPCPAPELLGGPSVFLFPPKPKDTLMISRTPEVTCVVVDVSHEDPEVKFNWYVDGVEVHNAKTKPREEQY**A**STYRVVSVLTVLHQDWLNGKEYKCKVSNKALPAPIEKTISKAKGQPREPQVYTLPPSRDELTKNQVSLTCLVKGFYPSDIAVEWESNGQPENNYKTTPPVLDSDGSFFLYSKLTVDKSRWQQGNVFSCSVMHEALHNHYTQKSLSLSPGK |
|  | Leader Ig heavy chain V-I region HG3, CD95(ed): CRD1, CRD2, CRD3, **Flag-tag**, cleavage site, heavy constant (**N297A**) |  |
| 6 | CD95ed-Flag-CL-pCR3 | MDWTWRVFCLLAVAPGAHSLEVARLSSKSVNAQVTDINSKGLELRKTVTTVETQNLEGLHHDGQFCHKPCPPGERKARDCTVNGDEPDCVPCQEGKEYTDKAHFSSKCRRCRLCDEGHGLEVEINCTRTQNTKCRCKPNFFCNSTVCEHCDPCTKCEHGIIKECTLTSNTKCKEEGSRSNGS**DYKDDDDK**EFEIKRTVAAPSVFIFPPSDEQLKSGTASVVCLLNNFYPREAKVQWKVDNALQSGNSQESVTEQDSKDSTYSLSSTLTLSKADYEKHKVYACEVTHQGLSSPVTKSFNRGEC |
|  | Leader Ig heavy chain V-I region HG3, CD95(ed): CRD1, CRD2, CRD3, **Flag-tag**, cleavage site, light constant |  |
| 7 | CD95ed-Flag-CL-GpL-pCR3 | MDWTWRVFCLLAVAPGAHSLEVARLSSKSVNAQVTDINSKGLELRKTVTTVETQNLEGLHHDGQFCHKPCPPGERKARDCTVNGDEPDCVPCQEGKEYTDKAHFSSKCRRCRLCDEGHGLEVEINCTRTQNTKCRCKPNFFCNSTVCEHCDPCTKCEHGIIKECTLTSNTKCKEEGSRSNGS**DYKDDDDK**EFEIKRTVAAPSVFIFPPSDEQLKSGTASVVCLLNNFYPREAKVQWKVDNALQSGNSQESVTEQDSKDSTYSLSSTLTLSKADYEKHKVYACEVTHQGLSSPVTKSFNRGECLEKPTENNEDFNIVAVASNFATTDLDADRGKLPGKKLPLEVLKEMEANARKAGCTRGCLICLSHIKCTPKMKKFIPGRCHTYEGDKESAQGGIGEAIVDIPEIPGFKDLEPMEQFIAQVDLCVDCTTGCLKGLANVQCSDLLKKWLPQRCATFASKIQGQVDKIKGAGGD |
|  | Leader Ig heavy chain V-I region HG3, CD95(ed): CRD1, CRD2, CRD3, **Flag-tag**, cleavage site, light constant, GpL |  |
| 8 | CD95ed-TNC-Fc(DANA)-2xFlag-pCR3 | *MLGIWTLLPLVLTSVARLSSKSVNA*QVTDINSKGLELRKTVTTVETQNLEGLHHDGQFCHKPCPPGERKARDCTVNGDEPDCVPCQEGKEYTDKAHFSSKCRRCRLCDEGHGLEVEINCTRTQNTKCRCKPNFFCNSTVCEHCDPCTKCEHGIIKECTLTSNTKCKEEGSDIACGCAAAPDIKDLLSRLEELEGLVSSLREQGTGELGTKTHTCPPCPAPELLGGPSVFLFPPKPKDTLMISRTPEVTCVVV**A**VSHEDPEVKFNWYVDGVEVHNAKTKPREEQY**A**STYRVVSVLTVLHQDWLNGKEYKCKVSNKALPAPIEKTISKAKGQPREPQVYTLPPSRDELTKNQVSLTCLVKGFYPSDIAVEWESNGQPENNYKTTPPVLDSDGSFFLYSKLTVDKSRWQQGNVFSCSVMHEALHNHYTQKSLSLSPGK**DYKDDDDK**EF**DYKDDDDK**LE |
|  | CD95(ed): *Leader*, CRD1, CRD2, CRD3, TNC, **Flag-tag**, cleavage site, hIgG1-Fc(**DANA**) |  |
| 9 | CD95ed-Flag-ALFA- pcDNA3.1+ | MDWTWRVFCLLAVAPGAHSLE*VARLSSKSVNA*QVTDINSKGLELRKTVTTVETQNLEGLHHDGQFCHKPCPPGERKARDCTVNGDEPDCVPCQEGKEYTDKAHFSSKCRRCRLCDEGHGLEVEINCTRTQNTKCRCKPNFFCNSTVCEHCDPCTKCEHGIIKECTLTSNTKCKEEGSRSNGS**DYKDDDDK**EFPSRLEEELRRRLTEP |
|  | Leader Ig heavy chain V-I region HG3, CD95(ed): CRD1, CRD2, CRD3, cleavage site, **Flag-tag**, ALFA |  |
| 10 | anti-ALFA-VHH-2xFlag-GpL-pCR3 | MNFGFSLIFLVLVLKGVQCEVKLVPRQLEVQLQESGGGLVQPGGSLRLSCTASGVTISALNAMAMGWYRQAPGERRVMVAAVSERGNAMYRESVQGRFTVTRDFTNKMVSLQMDNLKPEDTAVYYCHVLEDRVDSFHDYWGQGTQVTVSSGS**DYKDDDDK**EF**DYKDDDDK**LEKPTENNEDFNIVAVASNFATTDLDADRGKLPGKKLPLEVLKEMEANARKAGCTRGCLICLSHIKCTPKMKKFIPGRCHTYEGDKESAQGGIGEAIVDIPEIPGFKDLEPMEQFIAQVDLCVDCTTGCLKGLANVQCSDLLKKWLPQRCATFASKIQGQVDKIKGAGGD |
|  | Leader, anti-ALFA-VHH, **Flag-tag**, cleavage site, GpL |  |
| 11 | anti-ALFA-VHH-Fc-Flag-GpL-pCR3 | MNFGFSLIFLVLVLKGVQCEVKLVPRQLEVQLQESGGGLVQPGGSLRLSCTASGVTISALNAMAMGWYRQAPGERRVMVAAVSERGNAMYRESVQGRFTVTRDFTNKMVSLQMDNLKPEDTAVYYCHVLEDRVDSFHDYWGQGTQVTVSSGSKTHTCPPCPAPELLGGPSVFLFPPKPKDTLMISRTPEVTCVVVDVSHEDPEVKFNWYVDGVEVHNAKTKPREEQYNSTYRVVSVLTVLHQDWLNGKEYKCKVSNKALPAPIEKTISKAKGQPREPQVYTLPPSRDELTKNQVSLTCLVKGFYPSDIAVEWESNGQPENNYKTTPPVLDSDGSFFLYSKLTVDKSRWQQGNVFSCSVMHEALHNHYTQKSLSLSPGKEF**DYKDDDDK**LEKPTENNEDFNIVAVASNFATTDLDADRGKLPGKKLPLEVLKEMEANARKAGCTRGCLICLSHIKCTPKMKKFIPGRCHTYEGDKESAQGGIGEAIVDIPEIPGFKDLEPMEQFIAQVDLCVDCTTGCLKGLANVQCSDLLKKWLPQRCATFASKIQGQVDKIKGAGGD |
|  | Leader, anti-ALFA-VHH, cleavage site, hIgG1-Fc, **Flag-tag**, GpL |  |
| 12 | anti-ALFA-VHH-TNC-Flag-GpL-pCR3 | MNFGFSLIFLVLVLKGVQCEVKLVPRQLEVQLQESGGGLVQPGGSLRLSCTASGVTISALNAMAMGWYRQAPGERRVMVAAVSERGNAMYRESVQGRFTVTRDFTNKMVSLQMDNLKPEDTAVYYCHVLEDRVDSFHDYWGQGTQVTVSSGSDIACGCAAAPDIKDLLSRLEELEGLVSSLREQGTGEF**DYKDDDDK**LEKPTENNEDFNIVAVASNFATTDLDADRGKLPGKKLPLEVLKEMEANARKAGCTRGCLICLSHIKCTPKMKKFIPGRCHTYEGDKESAQGGIGEAIVDIPEIPGFKDLEPMEQFIAQVDLCVDCTTGCLKGLANVQCSDLLKKWLPQRCATFASKIQGQVDKIKGAGGD |
|  | Leader, anti-ALFA-VHH, cleavage site, TNC, **Flag-tag**, GpL |  |
